# Sibling species of the *Anopheles funestus* group, and their infection with malaria and lymphatic filarial parasites, in archived and newly collected specimens from northeastern Tanzania

**DOI:** 10.1186/s12936-015-0616-4

**Published:** 2015-03-06

**Authors:** Yahya A Derua, Michael Alifrangis, Stephen M Magesa, William N Kisinza, Paul E Simonsen

**Affiliations:** National Institute for Medical Research, Amani Research Centre, P.O. Box 81, Muheza, Tanga Tanzania; Centre for Medical Parasitology, Institute of International Health, Immunology, and Microbiology, University of Copenhagen, Copenhagen, Denmark; Global Health Division, RTI International, Dar es Salaam, Tanzania; Department of Veterinary Disease Biology, Section for Parasitology and Aquatic Diseases, University of Copenhagen, Copenhagen, Denmark

**Keywords:** *Anopheles funestus*, *Anopheles rivulorum*, *Anopheles parensis*, *Anopheles leesoni*, Malaria, Lymphatic filariasis, Tanzania

## Abstract

**Background:**

Studies on the East African coast have shown a recent dramatic decline in malaria vector density and change in composition of sibling species of the *Anopheles gambiae* complex, paralleled by a major decline in malaria incidence. In order to better understand the ongoing changes in vector-parasite dynamics in the area, and to allow for appropriate adjustment of control activities, the present study examined the composition, and malaria and lymphatic filarial infection, of sibling species of the *Anopheles funestus* group. Similar to the *An. gambiae* complex, the *An. funestus* group contains important vectors of both malaria and lymphatic filariasis.

**Methods:**

Archived (from 2005–2012) and newly collected (from 2014) specimens of the *An. funestus* group collected indoors using CDC light traps in villages in northeastern Tanzania were analysed. They were identified to sibling species by PCR based on amplification of species-specific nucleotide sequence in the ITS2 region on rDNA genes. The specimens were furthermore examined for infection with *Plasmodium falciparum* and *Wuchereria bancrofti* by PCR.

**Results:**

The identified sibling species were *An. funestus s.s.*, *Anopheles parensis, Anopheles rivulorum,* and *Anopheles leesoni,* with the first being by far the most common (overall 94.4%). When comparing archived specimens from 2005–2007 to those from 2008–2012, a small but statistically significant decrease in proportion of *An. funestus s.s.* was noted, but otherwise observed temporal changes in sibling species composition were minor. No *P. falciparum* was detected in archived specimens, while 8.3% of the newly collected *An. funestus s.s.* were positive for this parasite. The overall *W. bancrofti* infection rate decreased from 14.8% in the 2005–2007 archived specimens to only 0.5% in the newly collected specimens, and with overall 93.3% of infections being in *An. funestus s.s.*

**Conclusion:**

The study indicated that the composition of the *An. funestus* group had remained rather stable during the study period, with *An. funestus s.s.* being the most predominant. The study also showed increasing *P. falciparum* infection and decreasing *W. bancrofti* infection in *An. funestus s.s.* in the study period, most likely reflecting infection levels with these parasites in the human population in the area.

## Background

Malaria has remained an important mosquito-borne parasitic disease, which results in much human suffering and adversely affects socio-economic development in endemic countries. Despite renewed commitments to control malaria in the past decade, the World Health Organization estimated that 207 million cases of malaria, resulting in 627,000 deaths, occurred worldwide in 2012, of which 90% were in Africa [1]. In the African settings, malaria is mainly caused by the most virulent *Plasmodium falciparum* parasite, and transmitted by efficient mosquito vectors belonging to sibling species of the *Anopheles gambiae* complex and the *Anopheles funestus* group [2-6]. In Africa, sibling species of the same mosquitoes are also important vectors of the nematode parasite *Wuchereria bancrofti,* a widespread cause of disabling lymphatic filariasis [7].

Recent studies in northeastern Tanzania have documented a dramatic decline in the density of anopheline vectors, which has been reported to occur in parallel with a considerable decrease in prevalence of malaria [8-10]. The cause of the decline is not well understood and could not be directly linked to change in rainfall pattern or mosquito control intervention [9], and it occurred before the universal distribution of insecticide-impregnated bed nets in the area in 2011. However, it has substantially affected both the *An. gambiae* complex and the *An. funestus* group populations. Analysis of archived and newly collected *An. gambiae* complex specimens indicated a major simultaneous change in the composition of sibling species from being predominantly *An. gambiae sensu stricto* (*s.s.*) in the past to being predominantly *Anopheles arabiensis* [11]. While *An. gambiae s.s.* prefers to bite humans indoors, *An. arabiensis* has a less restricted behaviour and may feed indoors or outdoors and bite humans as well as other mammalian hosts [3,4,12]. The observed change in anopheline mosquito vectors could be due to environmental changes as well as mosquito control interventions [12-16].

The *An. funestus* group is comprised of nine sibling species of which *An. funestus s.s.* is the predominant, both in numbers and geographical distribution, and also the most anthropophilic [2,3,17]. As for *An. gambiae* complex, due to variation in their ecological requirements and behavior, accurate identification of the sibling species is crucial to understand their biology and hence parasite transmission capabilities [3]. In areas bordering northeastern Tanzania, four sibling species of the *An. funestus* group have been reported, namely *An. funestus s.s*., *Anopheles parensis, Anopheles rivulorum* and *Anopheles leesoni* [18]. Of these, *An. funestus s.s.* is the confirmed major vector for malaria and lymphatic filariasis while *An. rivulorum* has been implicated also to be a malaria vector [19-21]. Of particular interest to this study, the sibling species of the *An. funestus* group are known to exhibit species replacement and change in biting behaviour when confronted with insecticide-based interventions [22-26]. Following scale-up of distribution of insecticide-treated bed nets (ITNs) and environmental changes occurring in most of sub-Saharan Africa, and in view of the observed change in composition of *An. gambiae* complex, there is a need to investigate for a possible change in composition of the *An. funestus* group.

The current study focused on the *An. funestus* group by analysing newly collected and archived mosquito specimens. The availability of archived mosquitoes, collected from 2005 to 2012 in a study monitoring lymphatic filariasis elimination, provided a unique opportunity to compare past composition, and malaria and lymphatic filarial infection, in sibling species of the *An. funestus* group to that seen in freshly collected specimens from 2014.

## Methods

### Mosquito collection

Adult mosquitoes were sampled indoors in three villages in Tanga Region of northeastern Tanzania. These were Kirare (S 05.24943°; E 039.02876°) located approximately 20 km south of Tanga city, and the two neighbouring, Zeneti (S 05.22648°; E 038.66040°) and Kwakibuyu (S 05.26875°; E 038.66290°) located approximately 40 km west of Kirare. The villages have fairly similar topography and weather conditions and inhabitants practice subsistence farming and keep domestic animals, such as cattle, goats and chicken.

Kirare has been the site for an intensive study on lymphatic filariasis transmission and has maintained longitudinal mosquito surveillance since 2003. Throughout the period mosquitoes have been collected once weekly from 50 households using Center for Disease Control (CDC) light traps (John W Hock Co, Gainesville, FL, USA) as described previously [27-29]. In brief the traps were set at 19.00 hours and retrieved at 06.00 hours. Trapped mosquitoes were transported to the laboratory and sorted using morphological criteria. Live filarial vectors (*An. gambiae*, *An. funestus* and *Culex quinquefasciatus*) were knocked down with chloroform and dissected for *W. bancrofti* infection. Filarial vectors that were found dead in the traps between 2005 and 2012 were archived in Eppendorf tubes containing silica gel desiccant. Among these, 457 were sibling species of the *An. funestus* group and were included in the present study.

To establish the current composition and infectivity of the *An. funestus* group, adult mosquitoes were collected in Kwakibuyu and Zeneti in January and February 2014 (shortly after the short rains). Ten households were selected in each of the two villages for collection of mosquitoes for two weeks (14 consecutive nights) using CDC light traps as described above for Kirare. Collected sibling species of the *An. funestus* group (2,907 in total) were stored individually in perforated plastic ampoules. The ampoules were kept in sealable plastic bags containing silica gel desiccant for later PCR processing.

### DNA extraction from mosquitoes

DNA extraction was carried out by using the Bender buffer method as described elsewhere [11,30]. In brief, the method involved homogenizing individual mosquitoes in 100 μl Bender buffer (0.1 M NaCl, 0.2 M Sucrose, 0.1 M Tris–HCl (pH 7.5), 0.05 M EDTA (pH 9.1), 0.5% SDS) pre-heated at 65°C. Mosquito and parasite genomic DNA was then extracted and precipitated by potassium acetate and ethanol. The homogenate containing precipitated DNA was centrifuged to pellet the DNA. After removal of supernatant, the DNA pellet was air dried, reconstituted in sterile, double-distilled water and stored at −20°C until used for PCR amplifications.

### PCR for identification of *Anopheles funestus* group sibling species, and detection of *Plasmodium falciparum* and *Wuchereria bancrofti* infection

Sibling species of the *An. funestus* group were identified based on species-specific primers in the ITS2 region on the rDNA genes, a method previously developed to identify *An. funestus, Anopheles vaneedeni, An. rivulorum, An. leesoni,* and *An. parensis* [31]. Each PCR run was conducted in a final volume of 25 μl consisting of 0.5 μM of each of the six primers (An-fun UV, An-fun FUN, An-fun VAN, An-fun RIV, An-fun PAR, and An-fun LEES), 1:1 TEMPase Hot Start polymerase master mix (Ampliqon III, VWR - BieBerntsen, Denmark, including buffer containing MgCl_2_ and dNTPs, according to manufacturer’s instructions) and 3 μl of DNA extract. The samples were amplified in a VWR™ DuoCycler (VWR International bvba, Leuven, Belgium) and cycling conditions were 94°C for 15 min followed by 45 cycles of denaturation at 94°C for 30 sec, annealing at 50°C for 30 sec, extension at 72°C for 40 sec and final extension at 72°C for 10 min.

An aliquot of DNA from identified members of the *An. funestus* group was examined for infection with *P. falciparum* by utilizing a nested PCR as previously described [32]. In this PCR assay, only *P. falciparum* species-specific primers were used in the nested PCR as this is the most prevalent and important species in the study area. Outer PCR was run in a total volume of 20 μl containing 0.0625 μM of each of the two primers (PLU5 and PLU6), 1:1 Hot-Start TEMPase polymerase master mix and 2 μl of DNA extract. Following amplifications, the outer PCR products were used as a template in the nested PCR with *P. falciparum*-specific primers, where each 20 μl of PCR contained 0.25 μM of each of the two primers (rFAL1 and rFAL2), 1:1 Hot-Start TEMPase polymerase master mix and 1 μl of outer PCR product. Cycling conditions for the outer and nested PCR were as described previously [32].

An aliquot of DNA from identified sibling species of *An. funestus* group was moreover examined for *W. bancrofti* infection by PCR targeting highly repeated DNA sequences (the SspI repeat) found in *W. bancrofti* as previously described [33]. Each of the 20 μl of PCR consisted of 0.25 μM of each of the two primers (NV1 and NV2), 1:1 Hot-Start TEMPase polymerase master mix and 2 μl of DNA extract. The cycling conditions were as previously described [11].

Reference positive and negative controls were included in each batch of PCR runs to identify sibling species of the *An. funestus* group together with detection of *P. falciparum* and *W. bancrofti*. The resultant PCR products were separated based on their fragment size by gel electrophoresis and visualized under UV-light.

### Data analysis

Temporal change in sibling species composition and their infection rates with malaria and lymphatic filarial parasites was analysed with Pearson’s Chi-square test by using IBM SPSS Statistics (version 19), and p-values less than 0.05 were considered statistically significant.

### Ethical clearance

Meetings were held with village leaders (village executive officers, village chair persons and hamlet leaders) in the study villages to inform them about the study and to obtain their cooperation. Verbal consent was obtained from the heads of households before commencing mosquito collection in houses. Ethical approval for the study was provided by the Medical Research Coordinating Committee of the National Institute for Medical Research in Tanzania (Ref: NIMR/HQ/R.8a/Vol. IX/1563).

## Results

**Figure 1:**
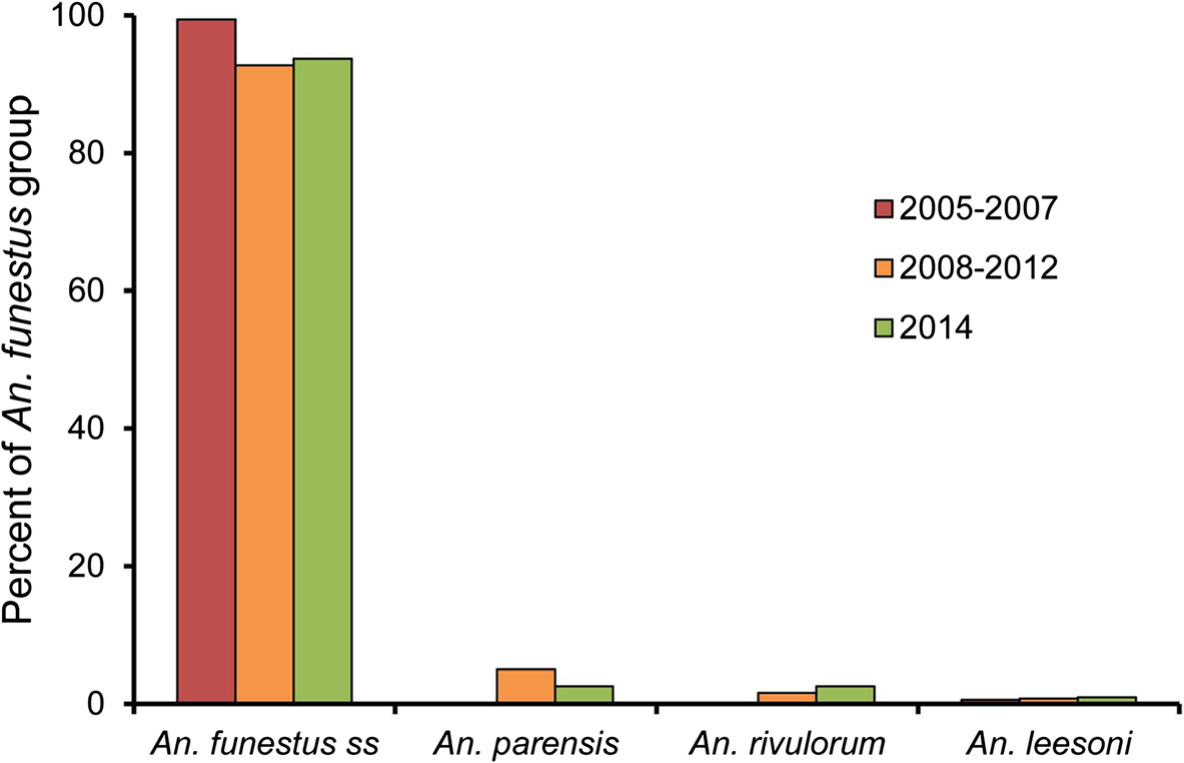
**Sibling species composition in the** ***Anopheles funestus*** **group at the three sampling periods.** Archived specimens from Kirare collected during 2005–2007 and 2008–2012; fresh specimens from Zeneti and Kwakibuyu collected in 2014.

A total of 1883 *An. funestus* group specimens were collected in Kirare during 2005–2012. Of these, 457 (24.3%) were archived and later processed for sibling species identification, namely 181 collected during 2005–2007 and 276 during 2008–2012 (Table 1). Of the processed specimens, 421 (92.1%) were successfully identified (89.5 and 93.8% for the two sampling periods, respectively). Among identified specimens, only *An. funestus s.s.* (99.4%) and *An. leesoni* (0.6%) were detected in the 2005–2007 collection, whereas *An. funestus s.s.*, *An. parensis, An. rivulorum,* and *An. leesoni* comprised 92.7, 5.0, 1.6 and 0.8% of the 2008–2012 collection, respectively (Table 1, Figure 1). Thus, a statistically significant decrease in proportion of *An. funestus s.s.* compared to the other sibling species combined was seen in the archived specimens when comparing the early to the later sampling period (n = 421; p = 0.002).

**Table 1:** **PCR testing for sibling species of the** ***Anopheles funestus*** **group and detection of** ***Plasmodium falciparum*** **and** ***Wuchereria bancrofti*** **infection**

**Collection village**	**Sampling period**	**No. included in test**	**PCR test for**	**No. positive PCR test#**	**No. (%) positive PCR tests according to sibling species**			
					***An. funestus*** **ss**	***An. parensis***	***An. rivulorum***	***An. leesoni***
Kirare	2005-2007*	181	Sibling species	162	161 (99.4)	0 (0.0)	0 (0.0)	1 (0.6)
			*P. falciparum*	0	0 (0.0)	-	-	0 (0.0)
			*W. bancrofti*	24	24 (14.9)	-	-	0 (0.0)
Kirare	2008-2012*	276	Sibling species	259	240 (92.7)	13 (5.0)	4 (1.6)	2 (0.8)
			*P. falciparum*	0	0 (0.0)	0 (0.0)	0 (0.0)	0 (0.0)
			*W. bancrofti*	18	15 (6.3)	1 (7.7)	1 (25.0)	1 (50.0)
Zeneti	2014**	460	Sibling species	459	428 (93.2)	13 (2.8)	13 (2.8)	5 (1.1)
			*P. falciparum*	42	42 (9.8)	0 (0.0)	0 (0.0)	0 (0.0)
			*W. bancrofti*	3	3 (0.7)	0 (0.0)	0 (0.0)	0 (0.0)
Kwakibuyu	2014**	116	Sibling species	116	111 (95.7)	2 (1.7)	2 (1.7)	1 (0.9)
			*P. falciparum*	6	6 (5.4)	0 (0.0)	0 (0.0)	0 (0.0)
			*W. bancrofti*	0	0 (0.0)	0 (0.0)	0 (0.0)	0 (0.0)

*) Archived specimens.

**) Newly collected specimens.

#) Only those identified to sibling species were tested for infection.

A total of 2,907 fresh *An. funestus* group specimens were collected from Zeneti (n = 2,791) and Kwakibuyu (n = 116) in 2014. Of these, 576 specimens were processed for sibling species identification, namely all 116 from Kwakibuyu and 460 randomly selected from Zeneti (Table 1). Of the processed specimens, 575 (99.8%) were successfully identified to species level. Among these the majority were *An. funestus s.s.* (93.7%), followed by *An. parensis* (2.6%), *An. rivulorum* (2.6%) and *An. leesoni* (1.0%) (Table 1, Figure 1). When comparing the two villages, there was no significant difference in proportion of *An. funestus s.s.* or the other sibling species combined between Kwakibuyu and Zeneti (n = 575; p = 0.3). Similarly, when all included, newly collected specimens from 2014 were compared to the archived specimens from 2008–2012, there was no significant difference in proportion of *An. funestus s.s.* or the other sibling species combined (n = 834; p = 0.7). The decrease in proportion of *An. funestus s.s.* between sampling period 2005–2007 and the later periods was mainly due to a relative increase in the *An. parensis* and *An. rivulorum* sibling species.

**Figure 2:**
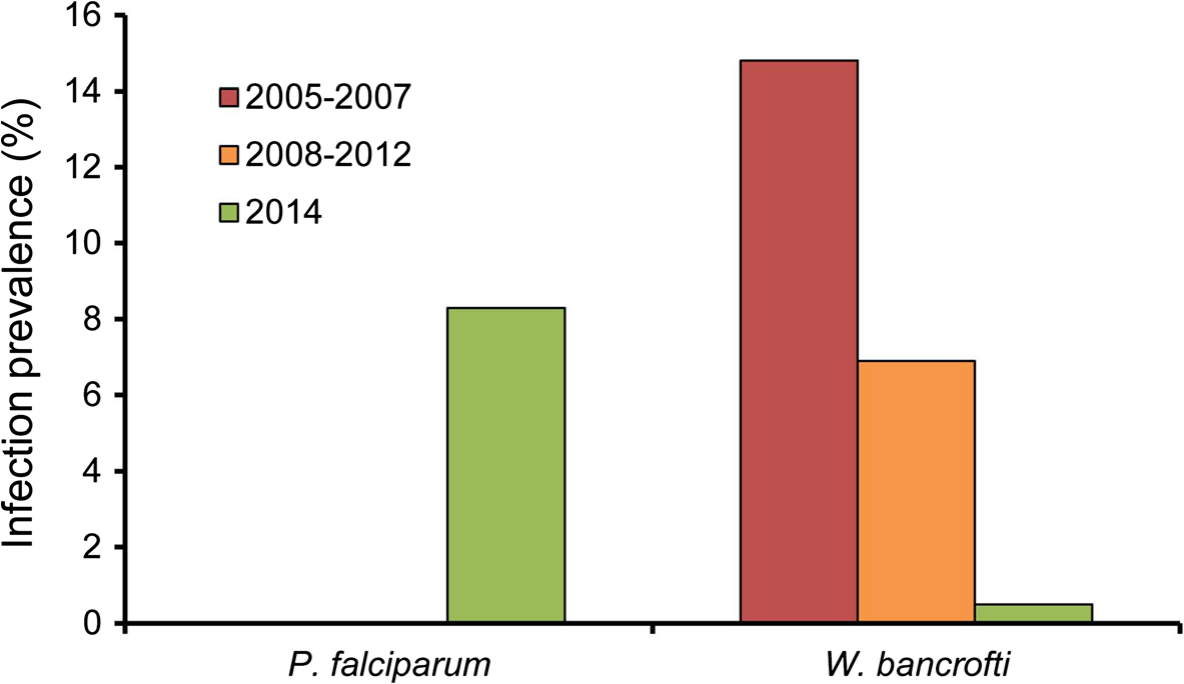
**Prevalence of** ***Plasmodium falciparum*** **and** ***Wuchereria bancrofti*** **infection in the sibling species of the** ***Anopheles funestus*** **group.** Archived specimens from Kirare collected during 2005–2007 and 2008–2012 survey periods; fresh specimens from Zeneti and Kwakibuyu collected in 2014.

Examination of the archived specimens for infection with *P. falciparum* (Table 1, Figure 2) showed no positives. However, 48 of the 575 newly collected specimens (8.3%) from 2014 were positive for *P. falciparum*, all of which were *An. funestus s.s.*

Examination of the archived specimens for *W. bancrofti* infection (Table 1, Figure 2) showed that 14.8% were positive in the 2005–2007 collection period. This decreased significantly to 6.9% in the 2008–2012 collection period (n = 421; p = 0.009). The trend of significant decrease continued to only 0.5% in the newly collected specimens from 2014 (n = 834; p <0.001). By far the majority of *W. bancrofti* infection was detected in *An. funestus s.s.* (42 of 45 cases, or 93.3%), but one of each of the other three sibling species were detected positive among the archived specimens.

## Discussion

Mosquitoes belonging to sibling species of the *An. gambiae* complex and *An. funestus* group are important vectors of malaria and lymphatic filariasis in northeastern Tanzania as well as in many other parts of sub-Saharan Africa [3,5,12,19,28,34,35]. However, these vectors have been shown to respond readily to environmental change, including insecticide-based interventions, such as indoor residual spraying with insecticide (IRS) and ITNs [12,15,22,36-39]. In northeastern Tanzania, a recent decline in overall malaria vector density affected both the *An. gambiae* complex and the *An. funestus* group [9]. Analysis of the composition of the *An. gambiae* complex revealed that the decline was most marked in *An. gambiae* s.s*.* and least for *An. arabiensis,* thus leading to predominance of the latter [11]. Other studies have indicated that change in population structure and behaviour of malaria mosquito vectors is not a new phenomenon [22,23,40]. With sibling species having different transmission potential, such changes are most likely to have important implications for the transmission and control of malaria and lymphatic filariasis.

The present study aimed at analysing changes in the composition and malaria and lymphatic filarial infection in sibling species of the *An. funestus* group. It took advantage of the availability of archived vector mosquitoes collected during 2005–2012 as part of a lymphatic filariasis study in Kirare village [27-29]. As the density of *An. funestus* group mosquitoes was extremely low in Kirare in 2014, fresh specimens were collected in the nearby villages of Kwakibuyu and Zeneti, which had similar topography and weather conditions but higher densities of these mosquitoes. All mosquitoes were collected indoors using CDC light traps and were analysed for species and infection status using PCR. Of 1,033 specimens analysed for species identity, 37 specimens, mainly from archived samples (n = 36), could not be identified using the current protocol. Following subsequent inclusion of *An. gambiae* complex oligonucleotide primers as described elsewhere [41], seven of the previously unidentified specimens were found to belong to the *An. gambiae* complex (four *An. arabiensis* and three *An. gambiae s.s.*). The remaining 30 unidentified specimens were not processed further. It should be noted that the malaria and lymphatic filarial parasite infections detected in the mosquitoes included all the vector-borne stages, since the PCR tests used were not designed to distinguish between infective and non-infective stages of *P. falciparum* and *W. bancrofti*.

The study revealed that *An. funestus s.s.*, *An. parensis, An. rivulorum,* and *An. leesoni* were the sibling species found in the study area, as reported previously [18], and that *An. funestus s.s.* was by far the most predominant. Thus, overall 94.4% of the identified sibling species were *An. funestus s.s.* Analysis of the archived specimens revealed a small but statistically significant decrease in the proportion of *An. funestus* group from the early (2005–2007) to the late (2008–2012) period. Analysis of newly collected specimens showed no statistically significant difference in proportion of *An. funestus s.s.* between the two neighbouring sampling villages. Moreover, there was no significant difference in proportion of *An. funestus s.s.* between the archived specimens collected in 2008–2012 and the newly collected specimens in 2014, even though the specimens were collected at slightly different locations (and therefore the comparison should be made with caution). The observed slight changes in sibling species composition over time are not likely to have much practical implication for parasite transmission and control unless they continue and reach larger magnitudes in the future.

The relatively stable proportion of *An. funestus s.s.* within the *An. funestus* group during the study period, while the proportion of *An. gambiae s.s.* within the *An. gambiae* complex had declined considerably, is of interest when it comes to the role of these sibling species in transmission of malaria and lymphatic filariasis. It was expected that since *An. funestus s.s.* has similar feeding and resting behaviour as *An. gambiae s.s.*, their response to, for example, change in environment (including applied control measures) would not differ much. However, the findings suggest that *An. funestus s.s.* is gradually overtaking *An. gambiae s.s.* as the most important vector for indoor malaria transmission in the study area. A similar situation has also been noted in the same area in relation to transmission of lymphatic filariasis, with *Cx. quinquefasciatus* gradually overtaking *An. gambiae* and *An. funestus* as the most important vectors [27]. A system like this, whereby one vector replaces another in response to control interventions and/or environmental changes, and thereby maintains transmission, intensifies the challenges of mosquito-borne disease control in Africa. The finding of high proportions of *An. funestus s.s*., known for their ferocity to enter houses to bite people, calls for assessment of their susceptibility to insecticides used for impregnating ITNs, as such nets are widely used in the study areas.

No *P. falciparum* infections were detected in archived specimens of the *An. funestus* group, probably due to a combination of low malaria transmission rate in the 2005–2012 collection period and the relatively low number of mosquitoes examined. On the contrary, the infection rate in the newly collected specimens from 2014 was quite high (8.3%) and corresponded closely to rates detected (10.2%) by Temu and colleagues [18] before the onset of decline in malaria prevalence. The study thus suggests that there had been a recent increase in human malaria parasitaemia. This finding corroborates well with observations by other researchers working in nearby villages indicating that recently the malaria prevalence has increased significantly after several years of continued decline (Deus Ishengoma, pers. comm.). All the *P. falciparum* infections were detected in *An. funestus s.s.* Although only few of the other sibling species were caught, and although *An. rivulorum* has been implicated as a malaria vector [19,21], the study confirmed that *An. funestus s.s.* was still the major malaria vector from this group in the area. Previous studies in both Kenya and Tanzania have indicated that the role of *An. funestus s.s.* as an important malaria vector is increasing [42,43].

*Wuchereria bancrofti* infection rates in the *An. funestus* group declined significantly in the archived specimens from 14.8% in the 2005–2007 to 6.9% in the 2008–2012 survey periods. The decline continued to only 0.5% in the newly collected specimens from 2014. It cannot be excluded that the archived mosquitoes may have had a slightly higher *W. bancrofti* infection rate than the newly collected ones because of inclusion of only dead mosquito specimens in earlier sampling period compared to the latter sampling which included both live and dead mosquito specimens in the traps. However, as the PCR test detected all stages of *W. bancrofti*, it is likely that the decline in vector infection rate was mainly a reflection of the marked decrease in human microfilarial prevalence occurring as a result of ongoing lymphatic filariasis control efforts in the area [27-29]. The study suggests that the *An. funestus* group plays an important role as vectors of *W. bancrofti*. However, the fact that all four sibling species of the *An. funestus* group were detected positive for this infection does not necessarily mean that they are also capable of supporting development of the filarial larvae to the infective stage. More studies are needed to elucidate the role of the individual sibling species in the transmission of lymphatic filariasis in northeastern Tanzania.

The study suggests that the factors which had recently significantly suppressed *An. gambiae s.s.* to rarity within the *An. gambiae* complex had not affected its vector cousin *An. funestus s.s.* to the same magnitude within the *An. funestus* group. This may have important implication for the epidemiology and control of malaria and lymphatic filariasis. *Anopheles funestus s.s.* breeds in permanent and semi-permanent water bodies and has the potential to be present as vector throughout the year, contrary to *An. gambiae s.s.* and *An. arabiensis* which prefers to breed in temporary, sunlit pools. The finding of substantial numbers of *An. funestus s.s.* indoors in areas where universal coverage with ITNs was implemented in 2011 calls for comprehensive investigations on bed-net use and insecticide susceptibility status of this mosquito vector.

## Conclusions

The study indicated that the sibling species composition of the *An. funestus* group in the study area had not changed much in recent years. Although a minor, but statistically significant, decline in *An. funestus s.s.* was observed, this sibling species was still by far the most dominant and had remained an important malaria and lymphatic filariasis vector. As measured by infection in mosquitoes, the findings confirmed that *W. bancrofti* infection in humans had declined considerably over the study period, while *P. falciparum* infection had increased in the area. The finding of a high proportion of *An. funestus s.s.* indoors in an area with universal ITN coverage calls for further investigations on bed-net coverage and susceptibility status of this vector to insecticides used for mosquito control.
